# Expression of prophage-encoded endolysins contributes to autolysis of *Lactococcus lactis*

**DOI:** 10.1007/s00253-016-7822-z

**Published:** 2016-09-22

**Authors:** Ganesh Ram R. Visweswaran, Dorota Kurek, Monika Szeliga, Francisco Romero Pastrana, Oscar P. Kuipers, Jan Kok, Girbe Buist

**Affiliations:** 1 Department of Molecular Genetics, Groningen Biomolecular Sciences and Biotechnology Institute (GBB), University of Groningen, Nijenborgh 7, 9747 AG Groningen, the Netherlands; 2 Department of Medical Microbiology, University of Groningen, University Medical Centre Groningen, Hanzeplein 1, 9700 Groningen, RB the Netherlands; 3 Department of Immunology, University of Oslo, Rikshospitalet, Sognsvannsveien 20, 0372 Oslo, Norway

**Keywords:** Autolysis, Bacteriophage, Endolysins, *Lactococcus lactis*

## Abstract

Analysis of autolysis of derivatives of *Lactococcus lactis* subsp. *cremoris* MG1363 and subsp. *lactis* IL1403, both lacking the major autolysin AcmA, showed that *L. lactis* IL1403 still lysed during growth while *L. lactis* MG1363 did not. Zymographic analysis revealed that a peptidoglycan hydrolase activity of around 30 kDa is present in cell extracts of *L. lactis* IL1403 that could not be detected in strain MG1363. A comparison of all genes encoding putative peptidoglycan hydrolases of IL1403 and MG1363 led to the assumption that one or more of the 99 % homologous 27.9-kDa endolysins encoded by the prophages bIL285, bIL286 and bIL309 could account for the autolysis phenotype of IL1403. Induced expression of the endolysins from bIL285, bIL286 or bIL309 in *L. lactis* MG1363 resulted in detectable lysis or lytic activity. Prophage deletion and insertion derivatives of *L. lactis* IL1403 had a reduced cell lysis phenotype. RT-qPCR and zymogram analysis showed that each of these strains still expressed one or more of the three phage lysins. A homologous gene and an endolysin activity were also identified in the natural starter culture *L. lactis* subsp. *cremoris* strains E8, Wg2 and HP, and the lytic activity could be detected under growth conditions that were identical as those used for IL1403. The results presented here show that these endolysins of *L. lactis* are expressed during normal growth and contribute to autolysis without production of (lytic) phages. Screening for natural strains expressing homologous endolysins could help in the selection of strains with enhanced autolysis and, thus, cheese ripening properties.

**Electronic supplementary material:**

The online version of this article (doi:10.1007/s00253-016-7822-z) contains supplementary material, which is available to authorized users.

## Introduction

A balanced action of peptidoglycan (PG) synthesis and degradation is essential for bacterial growth. To be able to grow and divide, bacteria express PG hydrolases that open up the rigid PG saccules (Vollmer et al. 2008). PG hydrolases play amongst others important roles in cell division and separation, cell wall turnover, PG modifications, sporulation, competence development, flagella formation and the activity of some antibiotics (Vollmer et al. 2008).

The glycan chains of PG can be cleaved by *N*-acetylglucosaminidases or *N*-acetylmuramidases. *N*-acetylmuramoyl-L-alanine amidases (amidases) hydrolyse the bond between the glycan chain and the pentapeptide side chain, while carboxypeptidases and endopeptidases can cleave the different bonds of the peptide side chain. For instance, DD-carboxypeptidases remove the terminal D-alanine residues of a pentapeptide to form a tetrapeptide thereby generating an acceptor for interpeptide bridge formation. Some endopeptidases cleave within the interpeptide bridge that can be present between two peptide side chains (Vollmer et al. 2008). Autolysis of cells is the result of the uncontrolled action of PG hydrolases when cell wall assembly and/or repair are inhibited (Vollmer et al. 2008). Because autolysins are potentially lethal enzymes, their activity has to be tightly regulated. Regulation can occur at the transcriptional and at the translational level, but it mostly operated at the posttranslational level such as by maturation of a preenzyme by the action of proteases or via controlled binding to the PG.

The gram-positive lactic acid bacterium *Lactococcus lactis* expresses four *N*-acetylglucosaminidases, namely AcmA, AcmB, AcmC and AcmD, that share a homologous active site domain (Buist et al. 1997; Huard et al. 2003, 2004; Visweswaran et al. 2013). AcmA is required for cell separation and is the major autolysin that is responsible for lysis during the stationary phase of growth of *L. lactis* (Buist et al. 1995). An *acmA* mutant of *L. lactis* MG1363 has been shown to completely lose autolysis activity under laboratory conditions, while overexpression of AcmA resulted in increased lysis (Buist et al. 1997; Steen et al. 2007, 2005a).

AcmD contributes to cell separation and autolysis although these actions are dependent on the presence of AcmA activity (Visweswaran et al. 2013). Inactivation of *acmB* revealed that AcmB of *L. lactis* might also be involved in autolysis since the mutant lysed to a lesser extent than its parent *L. lactis* MG1363. The effect was dependent on the presence of AcmA activity as no effect on autolysis of *acmB* was observed in an *acmAB* double mutant of *L. lactis* MG1363 (Huard et al. 2003). AcmD and AcmA both contain three C-terminal LysM sequences that are needed for peptidoglycan binding (Visweswaran et al. 2013). The presence of cell wall constituents like LTA, S-layer proteins or peptidoglycan modifications such as increased *O*-acetylation or reduced D-alanylation has been shown to inhibit binding of LysM and therefore prevent undesired enzymatic activity of AcmA (Steen et al. 2003; Veiga et al. 2007; Buist et al. 2008). *N*-acetylglucosamine deacetylation of PG has been shown to decrease autolysis in *L. lactis* (Meyrand et al. 2007). Degradation of AcmA by the membrane-located protease HtrA and/or the extracellular proteinase PrtP of *L. lactis* directly affects the degree of autolysis (Buist et al. 1998; Bosma et al. 2006).

Besides the genome-encoded PG hydrolases, the expression of endolysins can also contribute to (auto)lysis of lactococcal cells. Phage-encoded lysins function in the release of phages from the host cells (Vollmer et al. 2008). Such endolysins are generally co-expressed with holins that form pores in the cytoplasmic membrane of the host, thereby abolishing membrane potential and translocating the endolysin over the membrane (Young 2002).

*L. lactis* is one of the main bacterial species used in the production of cheese. One of the most important steps in cheese ripening is the release of intracellular proteolytic enzymes into the cheese matrix, which is a result of (auto)lysis of the lactococcal cells (Steen et al. 2007). Steen et al. (2007) have compared AcmB, AcmC, AcmD, endopeptidase YjgB and endolysin from prophages bIL309 and LytR from bacteriophage r1t with respect to their ability to lyse *L. lactis* cells (Steen et al. 2007). All PG hydrolases were active when expressed in *L. lactis*. The lysis effect of the activities of AcmB, AcmD and YjgB was dependent on the presence of AcmA. Food-grade overexpression of LytR from the producer strain in conjunction with a starter strain to make Gouda-type cheese resulted in increased lysis of both strains (Steen et al. 2007).

In this report, we investigated the question whether the increased lysis of *L. lactis* IL1403 compared to strain MG1363 under identical conditions of growth may be due to the presence of an extra lytic activity that was discovered by exclusion analysis of the predicted PG hydrolase content of both strains. The expression of the IL1403 gene for this extra lytic activity in MG1363 resulted in increased lysis of the overexpressing strain indicating that the PG hydrolase contributes to lysis.

## Materials and methods

### Bacterial strains, plasmids and growth conditions

The strains and plasmids used in this study are listed in Table 1. *L. lactis* was grown in M17 broth (Difco, Becton Dickinson, France) at 30 °C as standing cultures or on M17 agar. M17 was supplemented with 0.5 % glucose (GM17). Erythromycin and chloramphenicol (both from Roche, Mannheim, Germany) were added to concentrations of 5 μg/ml, when needed.

**Table 1:** Bacterial strains and plasmids used in this study

Strain or plasmid	Relevant phenotype or genotype	Reference or source
*L. lactis subsp. cremoris*		
MG1363	Lactose (Lac)^–^, Proteinase (Prt)^–^; plasmid-free derivative of NCDO712	Gasson (1983)
MG1363*acmAΔ1*	Derivative of MG1363 carrying a 701-bp* SacI-Spe*I deletion in *acmA*	Buist et al. (1995)
NZ9000	MG1363 *pepN::nisRK*	Kuipers et al. (1998)
NZ9000*acmAΔ1*	Derivative of NZ9000 carrying a 701-bp *Sac*I/*Spe*I deletion in *acmA*	Steen et al. (2007)
PA1001	MG1363 *pepN::nisRK*, allows nisin-inducible expression, *ΔacmA ΔhtrA*	Bosma et al. (2006)
NZ9700	Nisin-producing transconjugant containing the nisin-sucrose transposon Tn*5276*	Kuipers et al. (1993)
E8	Prt^+^ wildtype	Kok (1990)
E8	Prt^–^ wildtype	Kok (1990)
Wg2	Prt^+^ wildtype	Kok et al. (1985)
Wg2	Prt^–^ wildtype	Kok et al. (1985)
HP	Prt^+^ wildtype	Kok et al. (1985)
HP	Prt^–^ wildtype	Kok et al. (1985)
*L. lactis subsp. lactis*		
IL1403	Plasmid-free derivative of IL594, Lac^–^ Prt^–^	Chopin et al. (1984)
IL1403*acmA::*I*SS1*	IL1403with an ISS1 insertion in *acmA*	Steen et al. (2008)
IL1946	IL1403 cured of bIL285 via DCO deletion using plasmid pE194	Chopin et al. (1989)
IL2005	Partial deletion of bIL285 following the insertion of pE194; bIL286 is no more inducible; Em^r^	Institut National de la Recherche Agronomique (INRA collection)
IL6047	IL2005 cured of bIL309	INRA collection
pNG8048e	Derivative of pNZ8048 carrying Em^R^ marker and the nisin-inducible P_nisA_ promoter	Steen et al. (2007)
pNG*bil*	pNG8048e derivative carrying *pi149* from prophage bIL309	Steen et al. (2007)
pNG*bil::pi252*	pNG8048e derivative carrying *pi252* from prophage bIL285	This study
pNG*bil::pi305*	pNG8048e derivative carrying *pi305* from prophage bIL286	This study

### (Quantitative) polymerase chain reaction

Polymerase chain reactions (PCRs) were performed in a Mastercycler gradient (Eppendorf, Nijmegen, the Netherlands) by using Taq DNA polymerase or Expand DNA polymerase according to the instructions of the manufacturer (Roche). The primer pairs used in RT-qPCR for the detection of the endolysin genes and their messenger RNA (mRNA) transcripts of the bacteriophages bIL286, bIL285 and bIL309, respectively, are presented in Suppl. Table S1. RNA was isolated from *L. lactis* strains at the mid-exponential phase of growth by using High Pure RNA Isolation Kit (Roche) and according to the manufacturer’s protocol (Roche), followed by reverse trascription to generate complementary DNA (cDNA) by using MMLV Reverse Transcriptase (Fermentas GmbH, St. Leon-Rot, Germany). RT-qPCR was performed by using cDNA obtained from different strains, specific primers and SYBR Green RT-qPCR master mix as suggested by the manufacturer (Fermentas GmbH). The relative mRNA expression levels of different genes were obtained after normalizing to that of the housekeeping gene, the RNA polymerase alpha subunit (*rpoA*).

### Construction of plasmids for the induced expression of endolysins from bIL285 and bIL286

Plasmids for the expression of the endolysins encoded by the phages bIL285 and bIL286 were constructed by generating PCR fragments of the genes *pi252* and *pi305*, respectively (Phusion Hot Start II, Thermo Fisher Scientific, Wilmington, Delaware USA) by using *L. lactis* subsp. *lactis* IL1403 genomic DNA as a template (ZR Fungal/Bacterial DNA MiniPrep, Zymo Research, Irvine, CA, USA) with primers bil285F/bil286R and bil286F/bil286R. PCR fragment of *pi252* was digested with *Bsa*I to generate cloning overhangs and *Eco*RV to digest co-amplified *pi149* and *pi305* PCR fragments. PCR fragment *pi305* was digested with *Bsa*I, *Apa*I and *Afl*II restriction enzymes (New England Biolabs, Ipswich, MA, USA) to generate cloning overhangs and digest co-amplified *pi149* and *pi252* PCR fragments, respectively. Both digested PCR fragments were inserted into *Nco*I/*Hin*dIII-linearized vector pNZ8048e. PCR products were purified by using the High Pure PCR Purification Kit (Analytic Jena, Jena, Germany). Ligations were performed by using T4 DNA Ligase (New England Biolabs), and the resulting plasmids were transferred to electrocompetent *L. lactis* PA1001 as described before (Leenhouts and Venema 1993). All selected plasmids were checked by sequencing (Eurofins MWG Operon, Ebersberg, Germany).

### SDS-PAGE, zymogram and Western hybridization

Cell extracts and supernatant samples were prepared as described before (Buist et al. 1998). For cell fractionation, 25 ml of an overnight culture of *L. lactis* was subjected to centrifugation. The spent medium fraction was dialysed against several changes of demineralized water, lyophilized and dissolved in 800 μl of denaturation buffer [2 % dithiothreitol, 15 % sucrose, 3.8 % sodium dodecyl sulphate (SDS) (all *w*/*v*)]. The cell pellet was resuspended in 1 ml of 50 mM sodium phosphate buffer (pH 6.5) containing 100 mM NaCl, 550 mM sucrose, 5 mg/ml lysozyme and 50 U of mutanolysin and incubated for 1 h at 37 °C. In order to collect the cell wall fraction, the cell suspension was centrifuged at 5000*×g* for 15 min. Eight hundred microlitres of the supernatant fraction was dialysed, lyophilized and dissolved as described for the spent medium fraction. Protoplasts were resuspended in 1 ml of 50 mM sodium phosphate buffer (pH 6.5) containing 100 mM NaCl and subjected to sonication (six pulses of 15 s spaced 30 s apart on ice) with a Soniprep 150 Ultrasonic Disintegrator (MSE Scientific Instruments, Sussex, England). Unbroken cells were removed by centrifugation (5000*×g* for 15 min at 4 °C). The supernatant was centrifuged at 30,000*×g* for 30 min. The pellet (membrane fraction) was resuspended in 1 ml of denaturation buffer. The supernatant (cytoplasmic fraction) was dialysed, lyophilized and dissolved as described for the spent medium fraction.

The expression of the phage lysins from bacteriophages bIL285, bIL286 or bIL309 was induced with nisin by the addition of 1/1000 volumes of a supernatant of a culture of the nisin-producing strain *L. lactis* NZ9700 bearing the plasmid pNG8048e (Steen et al. 2007). PG-degrading activity was detected by a zymogram staining technique by using SDS-polyacrylamide (PAA) (10 or 12.5 %) gels containing 0.15 % (*w*/*v*) autoclaved, lyophilized *Micrococcus lysodeikticus* ATCC 4698 cells (Sigma-Aldrich, St. Louis, MO) or 0.3 % (*w*/*v*) *L. lactis* IL1403 autoclaved cells as a substrate, as described previously (Buist et al. 1995). To detect all peptidoglycan degradation activities of the endolysins, AcmA and its degradation products simultaneously in all samples, *M. lysodeikticus* cell wall fragments, were used in most cases. Renaturation of SDS-PAA gels were performed at pH 7.4. A prestained broad-range SDS-PAGE molecular mass marker of Bio-Rad Laboratories was used as a reference (Bio-Rad, Hercules, CA).

SDS-PAA gels (without cells) were stained with Coomassie brilliant blue (Bio-Rad). For Western hybridizations, proteins were transferred from SDS-PAA gels to polyvinylidene difluoride membranes (Roche) as described by Towbin et al. (1979). AcmA was detected with 1:5000-diluted polyclonal rabbit anti-AcmA active site antibodies (Steen et al. 2005b) and 1:5000-diluted HRP-conjugated anti-rabbit antibodies by using the ECL Western Blotting System and protocol (Amersham, Buckinghamshire, UK).

### Optical density measurements and enzyme activity assays

Overnight cultures of *L. lactis* in M17 were diluted 100-fold in prewarmed M17 at 30 °C, and the optical densities at 600 nm (OD_600_) were followed in time by using a Novaspec II spectrophotometer (Pharmacia Biotech AB, Uppsala, Sweden).

For a measure of cell lysis, the presence of intracellular X-prolyl dipeptidyl aminopeptidase (PepX) in culture supernatants was measured by using the chromogenic substrate Ala-Pro-*p*-nitroanilid (BACHEM Feinchemicalien AG, Bubendorf, Switzerland) as described before (Steen et al. 2008).

AcmA activity was visualized on GM17 agar plates containing 0.2 % (*w*/*v*) autoclaved, lyophilized *M. lysodeikticus* cells as halos around colonies after overnight growth at 30 °C (Buist et al. 1995).

### UV induction

For UV induction of prophages, cultures were grown until an OD_600_ between 0.2 and 0.6. The cells from 1 ml of culture were collected by centrifugation and resuspended in 1 ml of 1 mM MgSO_4_. The cell suspension was poured in a petri dish and exposed to UV light (260 nm) for 5 s. Subsequently, 1 ml of 2-fold concentrated GM17 containing 10 mM CaCl_2_ was added. Reduction of the OD_600_ was analysed in a microtiter plate reader (Molecular Devices Corporation, Menlo Oaks, CA) for 4 h.

## Results

### A cell wall hydrolytic activity of ∼30 kDa is present in *L. lactis* IL1403, but not in *L. lactis* MG1363

**Fig. 1:**
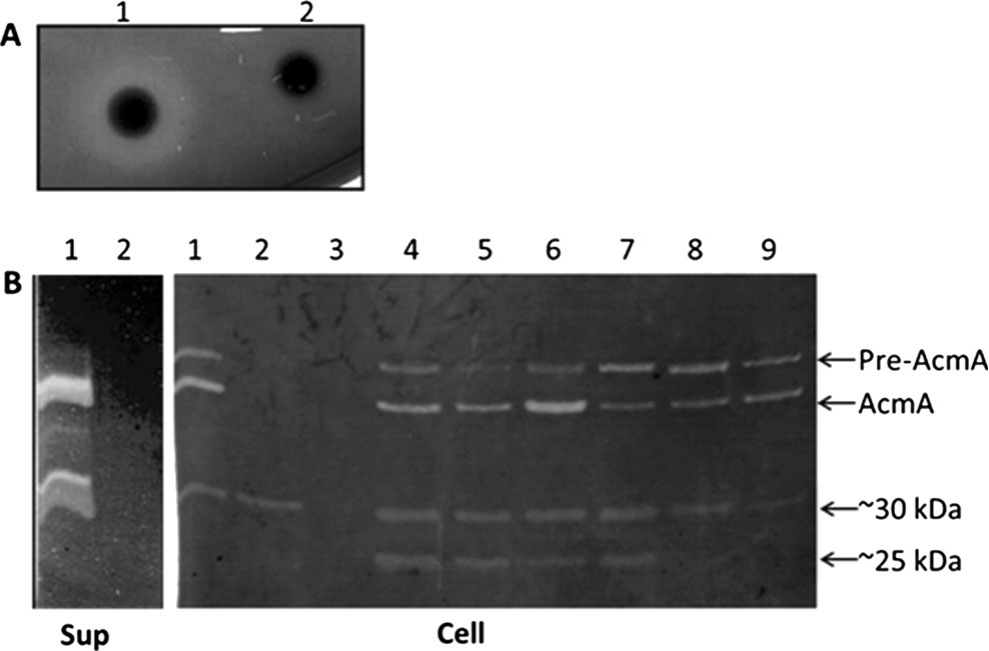
Cell wall hydrolytic activities of *L. lactis* IL1403 and IL1403*acmA*::IS*S1.* **a** Plate assay of *L. lactis* IL1403 (*1*) and *L. lactis* IL1403*acmA*::IS*S1* (*2*). **b** Zymographic analysis of supernatant (Sup) and cell fractions (Cell) of lactococcal strains *L. lactis* subsp. *lactis* IL1403 (Prt^−^) (*1*), *L. lactis* subsp. *lactis* IL1403*acmA*::IS*S1* (Prt^−^) (*2*), *L. lactis* subsp. *cemoris* MG1363*acmAΔ1* (Prt^−^) (*3*), *L. lactis* subsp. *cremoris* E8 (*4*), *L. lactis* subsp. *cremoris* E8 (Prt^−^) (*5*), *L. lactis* subsp. *cremoris* HP (*6*), *L. lactis* subsp. *cremoris* HP (Prt^−^) (*7*), *L. lactis* subsp. *cremoris* Wg2 (*8*) and *L. lactis* subsp. *cremoris* Wg2 (Prt^−^) (*9*). In both assays, 0.15 % *M. lysodeikticus* cell wall fragments was used as a substrate. Cell wall hydrolytic bands of (pre-)AcmA and the additional activities of ∼30 and ∼25 kDa are indicated in the right margin

Autolysis, as measured by the release of the intracellular X-prolyl dipeptidyl aminopeptidase activity into the culture supernatant, of *L. lactis* subsp. *cremoris* MG1363 and subsp. *lactis* IL1403 was examined during an incubation period of 78 h under identical conditions. This comparison revealed that the cells of strain IL1403 lysed to a higher extent than those of strain MG1363. Comparing the autolytic behaviour of the respective isogenic *acmA* mutants, by using a similar approach, showed that the *acmA* mutant of *L. lactis* IL1403 still lysed to some extent while the *L. lactis* MG1363 *acmA* mutant did not (Steen et al. 2008). The mutation in *acmA*, the gene for the major lactococcal autolysin, had been obtained by an internal deletion in MG1363 and by an IS*S1* transposon insertion in *acmA* of IL1403, and both strains had lost the AcmA PG hydrolase activity (Buist et al. 1995; Steen et al. 2008). In a plate assay for the detection of cell wall hydrolase activities by using *M. lysodeikticus* cell wall fragments as a substrate, no halo could be detected around colonies of the *acmA* insertion mutant of strain IL1403 (Fig. 1a). Zymographic analysis by using *M. lysodeikticus* or *L. lactis* cell wall fragments as a substrate showed that cell extracts of *L. lactis* IL1403*acmA*::IS*S1* contain a lytic activity of around 30 kDa which is not present in the supernatant fraction when renaturation was performed at pH 7.4 (Fig. 1b). In neither of these fractions of *L. lactis* MG1363*acmAΔ1* could a similar activity be detected (see lane 3 in Fig. 1b for the cell extract of MG1363). The band of ∼30 kDa appeared after prolonged incubation and was always more intense when lactococcal cell instead of *M. lysodeikticus* wall fragments was used as a substrate (results not shown). The fact that the ∼30-kDa lytic activity is still produced by the *L. lactis* IL1403 *acmA* mutant shows that it is not a degradation product of AcmA.

Zymographic analysis of cell and supernatant fractions of the *L. lactis cremoris* strains E8, HP and Wg2 and their PrtP protease-negative derivatives revealed that all three strains also contain an additional lytic activity of around 30 kDa (Fig. 1b). Cells of strains E8 and HP also contain a cell wall hydrolase activity of approximately 25 kDa. The fact that the lytic activities are also detectable in the PrtP-negative derivatives indicates that they are not degradation products of AcmA, which is degraded by PrtP (Buist et al. 1998). The detected bands might be degradation products of AcmA caused by the extracellular surface protease, HtrA, albeit that those products have mostly been detected in supernatant fractions of *L. lactis* MG1363 and they were hardly able to bind to the cell walls of whole cells (Bosma et al. 2006; Steen et al. 2005a). The lytic activities of ∼30 and ∼25 kDa were only detected in the cell fractions.

**Fig. 2:**
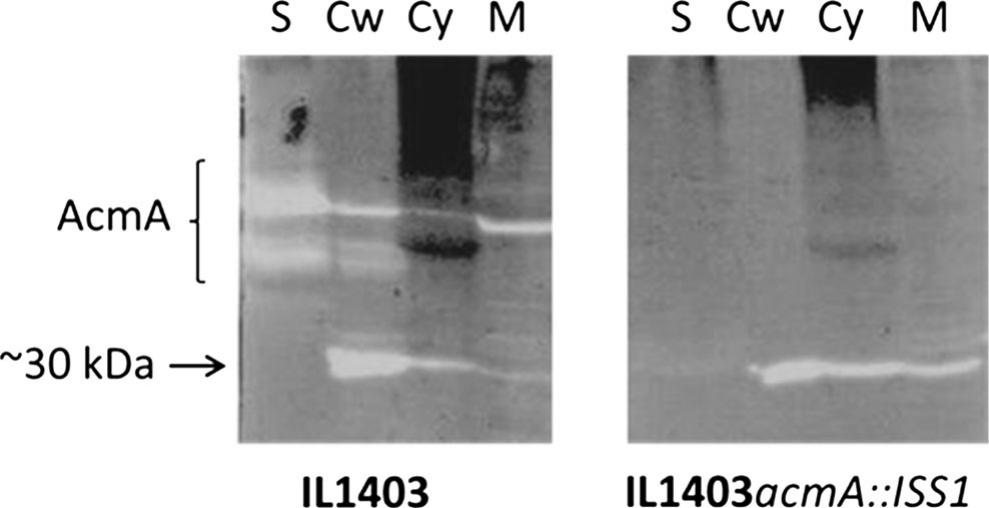
Localization of cell wall hydrolytic activities in *L. lactis* strains IL1403 and IL1403*acmA*::IS*S1*. Proteins of cytoplasmic (Cy), membrane (M), cell wall (Cw) and supernatant (S) fractions of both strains were prepared from overnight cultures and separated in a 12.5 % SDS-PAA gel as described in the Materials and Methods section. The gel contained 0.3 % (*w*/*v*) autoclaved cells of *L. lactis* IL1403 as a substrate. The various activity bands of (pre-)AcmA and their degradation products and the additional activity of ∼30 kDa are indicated

The ∼30-kDa cell wall hydrolase activity is present in the cytoplasm, the membrane and the cell wall of *L. lactis* IL1403 and *L. lactis* IL1403*acmA*::IS*S1*, but not in the supernatant (Fig. 2). The highest activity was observed in the cell wall fraction. No processing of the cell wall hydrolase activity, as observed for pre-AcmA (see Fig. 1b), could be detected, suggesting that the enzyme lacks a signal peptide for secretion via the general Sec machinery (Buist et al. 2006).

### Genes for (predicted) PG hydrolases of *L. lactis* strains IL1403 and MG1363

*L. lactis* has been suggested to possess 22 genes for putative PGHs based on PG hydrolase activity and cell wall binding domains (Layec et al. 2008). Literature searches and blast analyses showed that *L. lactis* IL1403 contains at least 18 genes for PG hydrolases (Table 2). From earlier studies, it is known that *L. lactis* MG1363 expresses the glucosaminidases AcmA, AcmB, AcmC and AcmD and the putative gamma-D-glutaminyl-L-lysyl-endopeptidase YjgB (Buist et al. 1995; Huard et al. 2004; Visweswaran et al. 2013). The genes for all five enzymes are present in the genome of IL1403 (Bolotin et al. 1999). BlastP analysis revealed that a second endopeptidase (encoded by *ypcD*) with an NlpC/P60 domain (Pfam PF00877) is present. The genome of IL1403 contains four genes (*acmB*, *usp45*, *yfcF*, *yqeC*) encoding putative amidases with a CHAP (cysteine, histidine-dependent amidohydrolases/peptidases) domain (Pfam PF05257). The gene *dacB* of MG1363 encodes a protein with L,D-carboxypeptidase activity that is involved in PG maturation (Courtin et al. 2006). Another D-Ala-D-Ala carboxypeptidase (DacA) of MG1363 converts a pentapeptide side chain in PG to a tetrapeptide side chain (Deghorain et al. 2010). Both *dacA* and *dacB* are present in IL1403. The *L. lactis* IL1403 genome contains four prophage-encoded lysin genes of which three homologues are encoded by the P335 phages bIL285, bIL286 and bIL309 (Chopin et al. 2001). The three phage lysins belong to the NLP/P60 family (pfam00877), contain an N-terminal active site domain and a C-terminal putative PG binding domain (pfam01471) (Labrie et al. 2004) and lack a predicted signal peptide for Sec-mediated secretion (Table 2). The comparison of these phage lysins shows that all are predicted to be 28-kDa proteins, are nearly identical and are homologous to the endolysin (*N*-acetylmuramoyl-L-alanine amidase) of phage BK5-T of *L. lactis* (Chopin et al. 2001). The fourth prophage encodes an endolysin with lysostaphin activity and a molecular mass of 99.4 kDa. These prophages are not present in the genome of MG1363 (Table 2, Wegmann et al. 2007). Blast analysis revealed that only the gene *llmg_2005* (YP_001033263) encodes a protein that is homologous to the C-terminal part of the bIL lysins.

**Table 2:** Genes for putative PG hydrolases in the genome of *L. lactis* IL1403

PGH family	Gene^a^	NCBI^b^	MM^c^	pI^d^	SP^e^	Reference or remark
	*acmA*	NP_266428	46.6 (40.3)	10.2 (9.9)	57–58 VQA-AT	Steen et al. (1995) and Steen et al. (2005b)
*N*-acetylglucosaminidase (together with cysteine,histidine-dependent amidohydrolases/peptidase domain in AcmB)	*acmB*	NP_268064	52.2 (48.8)	5.0 (4.6)	31 32 GVA-GH	Huard et al. (2003)
	*acmC*	NP_267521	23.7 (18.7)	9.4 (6.1)	41–42 NQA-QK	Huard et al. (2004)
	*acmD*	NP_266697	37.5 (34.9)	4.3 (3.9)	26–27 AQA-AS	Visweswaran et al. (2013)
Endo-β-*N*-acetylglucosaminidase	*ypcD*	NP_267648	61.2 (57.2)	7.3 (5.2)	37–38 VKA-NY	Layec et al. (2008)
Transglycosylase	*yrbB*	NP_267818	20.5 (17.9)	5.2 (4.7)	26–27 AHA-DT	Layec et al. (2008)
D-Ala-D-Ala carboxypeptidase	*dacA*	NP_268420	46.9 (44.3)	6.9 (6.6)	25–26 VSA-AT	Deghorain et al. (2010)
l-Lys-d-Ala carboxypeptidase	*dacB*	NP_267106	27.5 (24.0)	6.1 (5.4)	31–32 KSN-ST	Courtin et al. (2006)
γ-D-glutamyl-L-lysyl endopeptidase	*yjgB*	NP_267092	20.9 (17.6)	5.6 (4.9)	32–33 AKA-DS	Huard et al. (2004; Redko et al. (2007; Steen et al. (2007)
Cysteine, histidine-dependent amidohydrolase/peptidase	*usp45*	NP_268386	47.0 (44.2)	8.3 (6.4)	27–28 VYA-DT	Redko et al. (2007)
	*yfcF*	NP_266694	32.1 (26.7)	9.2 (6.8)	47–48 VQA-ST	Redko et al. (2007)
	*yqeC*	NP_267755	19.5	9.5	n.i.; SP present in MG1363 YqeC	Wrong start codon and deletion in IL1403; Redko et al. (2007)
Muramoyltetrapeptide carboxypeptidase	*yrgH*	NP_267869	36.4	5.2	n.i.	Wegmann et al. (2007)
	*pi252* (bIL285)	NP_267215	27.9	9.3	n.i.	Chopin et al. (2001)
*N*-acetylmuramyl-L-alanine amidase	*pi149* (bIL309)	NP_266640	27.9	9.4	n.i.	Chopin et al. (2001)
	*pi305* (bIL286)	NP_267535	27.9	9.2	n.i.	Chopin et al. (2001)
	*ygeA*	NP_266809	40.7	8.8	n.i.	Wrong start codon; Bolotin et al. (1999)
Lysostaphin	*pi244*	NP_267207	99.4	6.4	n.i.	Wegmann et al. (2007)

*n.i.* not identified

^a^Gene name as indicated in NCBI; genes also present in the genome of *L. lactis* MG1363 are indicated with a grey background

^b^NCBI reference numbers of which the prophage-encoded genes are indicated with a grey background

^c^Predicted molecular mass of the expressed and secreted (between brackets) proteins in kDa

^d^Calculated iso-electric points of the (secreted) proteins

^e^Predicted signal peptide cleavage sites by using www.cbs.dtu.dk/services/SignalP/ or bmbpcu36.leeds.ac.uk/prot_analysis/Signal.html

### The ∼30-kDa cell wall hydrolase activity in *L. lactis* IL1403 is phage encoded

The comparison of the molecular masses of the predicted PG hydrolases of IL1403 suggests that *acmD*, *yfcF*, *yrgH*, *dacB* or the endolysin genes *pi252* (phage bIL285), *pi149* (phage bIL309) or *pi305* (phage bIL286) could all be responsible for the detected ∼30-kDa PG hydrolytic activity. As *acmD*, *yfcF* and *dacB* genes are also present in the genome of *L. lactis* MG1363 (Wegmann et al. 2007 and Table 2), it is unlikely that the ∼30-kDa activity is encoded by these genes. As *L. lactis* MG1363 AcmD could only be detected in zymography by using a renaturation buffer of pH of 4 (Huard et al. 2004; Visweswaran et al. 2013), while the ∼30-kDa activity was detectable at pH 7.4, the detected activity cannot be AcmD. Carboxypeptidases such as *yrgH* do not damage to the integrity of the PG to the extent that they cause autolysis, and thus, *yrgH* most likely does not specify the ∼30-kDa lytic activity.

**Fig. 3:**
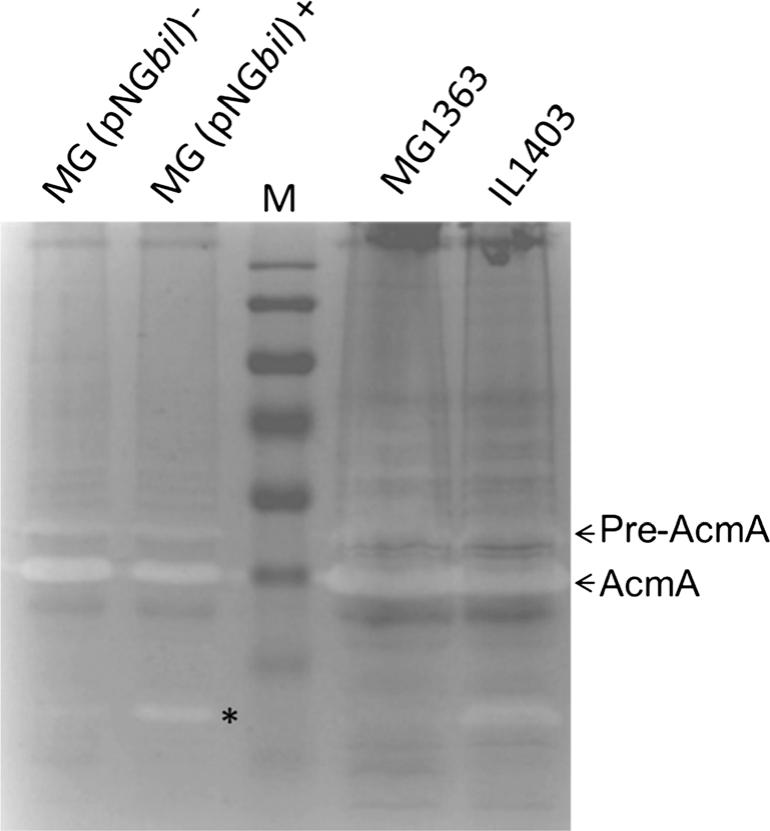
Zymographic analysis of the cell wall hydrolase activity of the endolysin of phage bIL309. Cell-free extracts of *L. lactis* MG1363 (MG), *L. lactis* IL1403 (IL) and *L. lactis* MG1363 (pNG*bil*) from either nisin-induced (+) or uninduced (−) cultures were separated in a 10 % PAA gel which was subsequently renatured to detect the lytic activities. Bands of lytic activity of (pre-)AcmA and bIL309 endolysin (*asterisks*) are indicated. The gel contained 0.15 % autoclaved, lyophilized *M. lysodeikticus* cells as a substrate

Based on this exclusion, the endolysins of bIL285, bIL286 or bIL309 could encode the detected activity. Previously, it was shown that expression of the lysin of bIL309 in *L. lactis* MG1363 resulted in increased lysis, but the molecular mass of the lysin and its cellular location were not investigated in this study (Steen et al. 2007). Here, we show that nisin-induced lysin of phage bIL309 in *L. lactis* NZ9000 (a derivative of MG1363) carrying plasmid pNG*bil* is only present in the cell fraction and that the active enzyme has a molecular mass of ∼30 kDa (Fig. 3). This result indicates that the PG hydrolase activity of ∼30 kDa in *L. lactis* IL1403 is a phage lysin and has a predicted molecular mass and pI of 27.9 and 9.2, respectively (Table 2). Because of the fact that the size of this endolysin is identical to that of the lysins of the phages bIL285 and bIL286, it is also possible that one or a combination of these enzymes is expressed in IL1403.

Induced expression of the phage endolysins from phages bIL285 and bIL286 in the *L. lactis* MG1363-derived *acmA htrA* double mutant showed that these proteins are mainly present in the cell fraction. Although the uninduced PA1001 strains did not lyse, lysis was observed for the induced strains expressing either of the endolysins as cellular proteins are present in the supernatant fractions of these strains (Suppl. Fig. S1). Because an ∼30-kDa lytic activity was also detected in the *L. lactis* subsp. *cremoris* strains E8, HP and Wg2, a PCR on their chromosomal DNAs was performed by using primers specific for the endolysin genes of phages bIL285, bIL286 and bIL309 of IL1403 (Suppl. Table S1). PCR products of equal sizes were obtained for E8, HP, Wg2 and IL1403 indicating that the same endolysin genes are present in all four strains. Blast analysis by using the amino acid sequences of the endolysins encoded by pi252, pi305 or pi149 against the draft genome sequence of *L. lactis* subsp. *cremoris* HP (Lambie et al. 2014) showed that it encodes one endolysin with 96 % identity (GenBank EUN34905). As expected, when chromosomal DNA of MG1363 was used as a template, no PCR product was obtained (results not shown).

These results show that a ∼30-kDa PG hydrolase activity is encoded by one or more endolysin genes in *L. lactis* IL1403 and is expressed during exponential growth.

### bIL phages of *L. lactis* IL1403 are responsible for UV-induced lysis

**Fig. 4:**
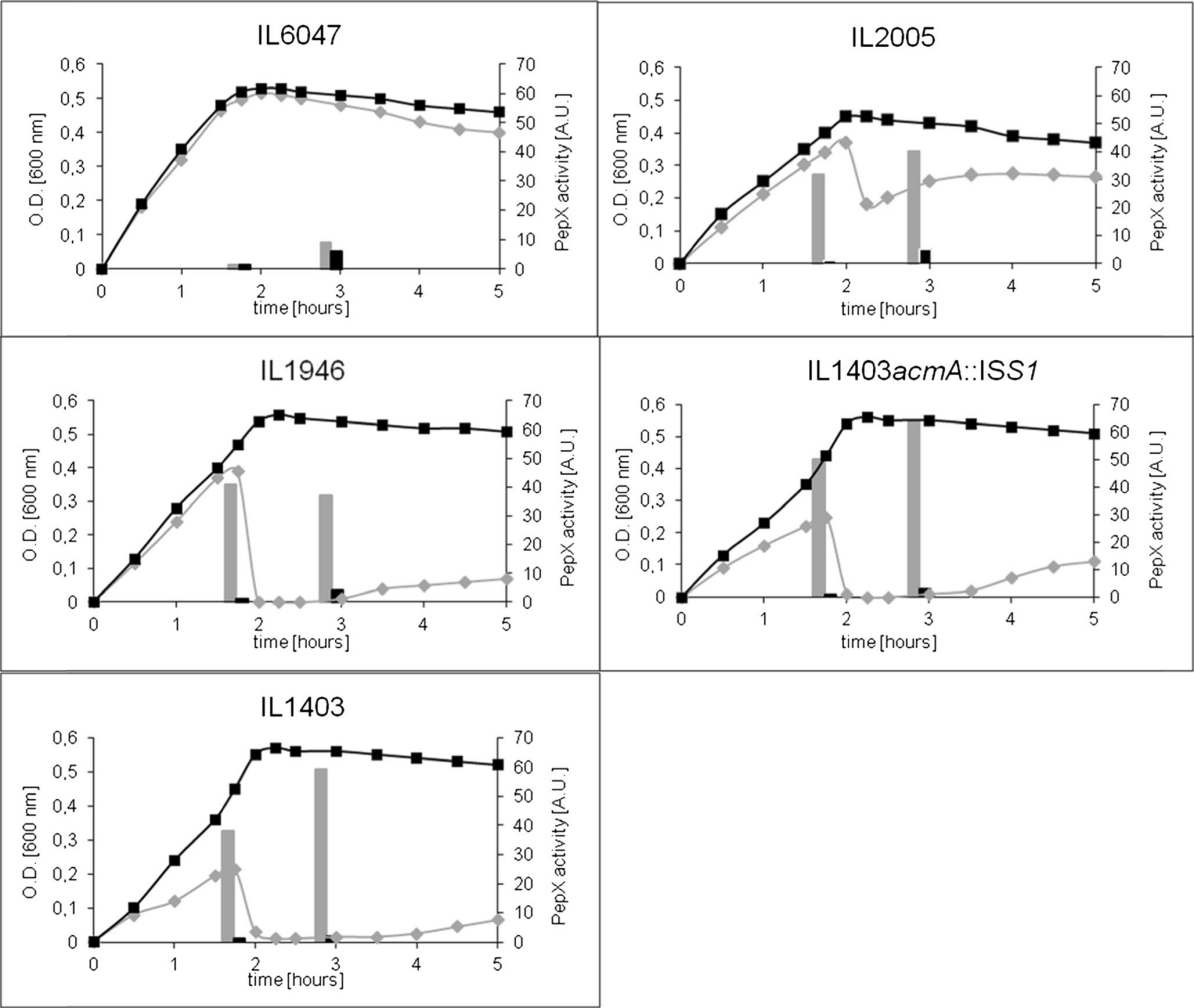
Growth and lysis of *L. lactis* IL1403 and its prophage mutants. Growth of *L. lactis* strains IL6047, IL2005, IL1946, IL1403*acmA*::IS*S1* and IL1403 was determined by measuring the OD_600_ (*left Y-axis*). Autolysis after treatment with (*grey bars*) or without (*black bars*) UV was determined by measuring the release of the intracellular peptidase PepX (*bars*; in arbitrary units) into the culture supernatants (*right Y-axis*)

**Fig. 5:**
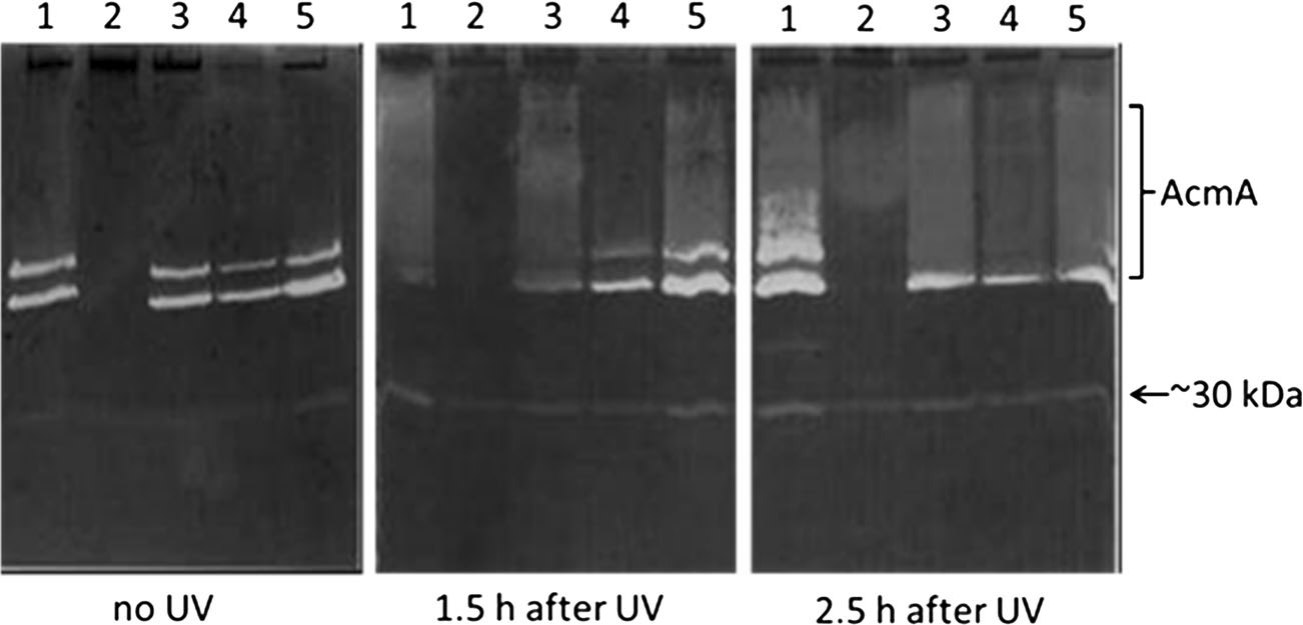
Activity of AcmA and the endolysin of phage bIL309 before or after phage induction by UV treatment. Cell-free extracts of *L. lactis* IL1403 (*1*), IL1403*acmA*::IS*S1* (*2*) and the isogenic prophage mutants *L. lactis* IL1946 (*3*), IL2005 (*4*) and IL6047 (*5*) were loaded on to a 12.5 % SDS-PAA gel containing 0.15 % autoclaved, lyophilized *M. lysodeikticus* cells as a substrate. Bands of lytic activity of (pre-)AcmA and the ∼30-kDa activity are indicated

To investigate the contribution of the various endolysins in lysis of *L. lactis* IL1403, derivatives of IL1403 were used that lack parts of or the complete prophages bIL285, bIL286 and/or bIL309. *L. lactis* IL1946 is a derivative of IL1403 that was cured of bIL285 via insertion and excision of plasmid pE194 (Chopin et al. 1989). *L. lactis* IL2005 has partially lost phage bIL285 following the insertion of pE194, while prophage bIL286 is no longer inducible due to the insertion of an erythromycin resistance gene. *L. lactis* IL6047 is a derivative of strain IL2005 that has been cured of phage bIL309. The prophages of *L. lactis* IL1403 and its derivatives were induced through exposure to UV light. Strains IL1403, IL1403*acmA*::IS*S1* and IL946 behaved similarly with respect to reduction of the OD_600_ and the release of the cytoplasmic peptidase PepX into the culture supernatant (Fig. 4). Although the reduction in OD_600_ of *L. lactis* IL2005 was less, the release of PepX is nearly similar. No lysis was observed of *L. lactis* IL6047, which lacks the prophage bIL309, while bIL285 is partly deleted and bIL286 is not inducible (Fig. 4). The release of PepX under uninduced conditions was nearly the same for all strains (Fig. 4). Zymographic analysis showed that more phage lysin activity is present in the cell fractions of all strains upon UV treatment (Fig. 5). Endolysin activity is also detectable in *L. lactis* IL6047 (Fig. 5). As no phages are produced by this strain, this likely reduces cellular lysis (Fig. 5).

These results shown that the prophage bIL285, bIL286 and bIL309 contribute to cellular lysis upon phage induction by UV but also that phage lysins are expressed without UV induction.

### Transcription of prophage lysin genes during normal growth of *L. lactis* IL1403

**Fig. 6:**
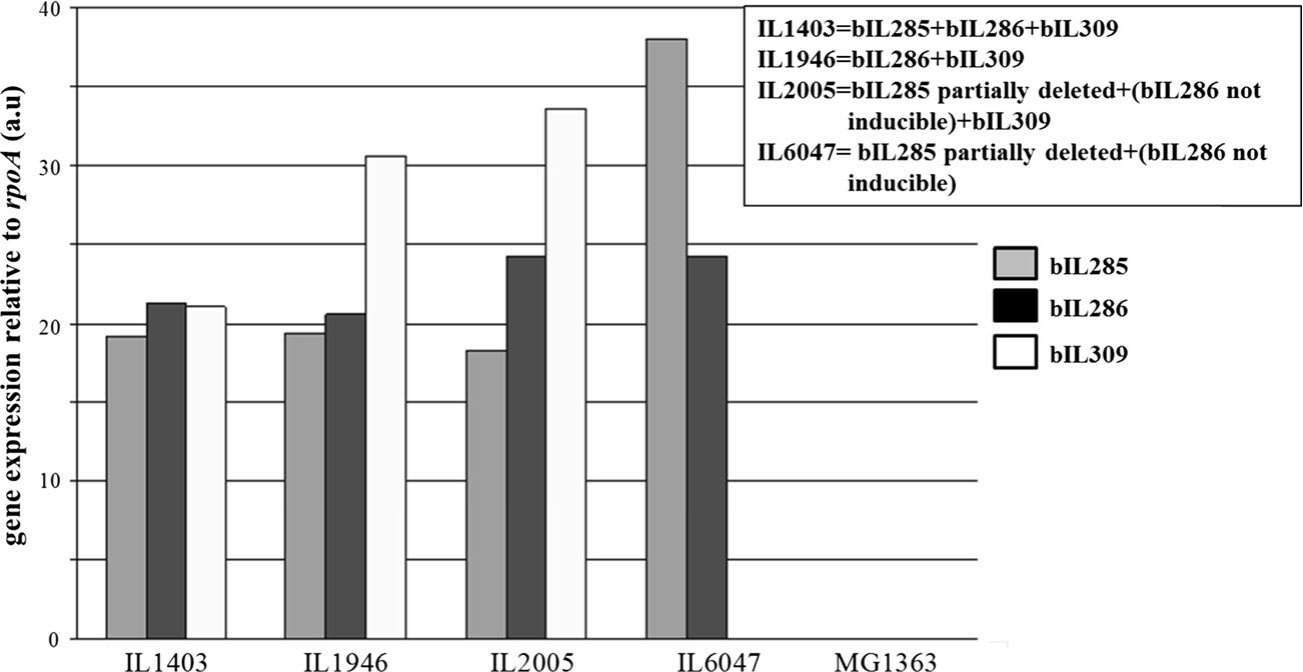
Transcription of endolysin genes of bIL285 (*grey bars*), bIL286 (*black bars*) and bIL309 (*white bars*) in *L. lactis* IL1403 and its prophage mutants. RNA was isolated from *L. lactis* strains IL1403, IL1946, IL2005, IL6047 and MG1363 after overnight growth in GM17 at 30 °C. RT-qPCR was done with primers specific for each of the endolysin genes (Suppl. Table S1). Relative mRNA expression levels of different genes, normalized to that of the housekeeping gene for the RNA polymerase alpha subunit (*rpoA*), are indicated on the *Y*-axis

Detection of endolysin activity in cell extracts from exponential phase cells of *L. lactis* IL1403 implies that their genes are transcribed during growth. A RT-qPCR was performed to verify this assumption on RNA samples taken at mid-exponential phase from cultures of *L. lactis* IL1403 and its prophage mutant derivatives by using endolysin gene-specific primers (Suppl. Table S1). In *L. lactis* strains IL1403, IL1946 and IL2005, PCR products were obtained for all three endolysin genes (Fig. 6). This was not anticipated for IL1946 as it was reported to lack the bIL285 phage and its endolysin gene (Chopin et al. 1989). No transcript for the endolysin gene of bIL285 was seen in *L. lactis* IL6047, as expected (Fig. 6). These results show that the endolysin genes of the prophages bIL285, bIL286 and bIL309 are expressed during growth in GM17 although lysis as a consequence of UV-induced prophage production was not observed.

## Discussion

In this paper, we show that *L. lactis* subsp. *lactis* IL1403 expresses a 27.9-kDa cellular and cell wall-located endolysin activity that is encoded by the phage lysin genes *pi252*, *pi305* and *pi149* of the prophages bIL285, bIL286 and bIL309, respectively. The endolysins contribute to autolysis of *L. lactis* subsp. *cremoris* MG1363, which lacks these prophages or homologous endolysin genes, lysed to a greater extent when the endolysin of phage bIL309 was overexpressed; zymographic analysis showed that this activity is similar in size to the 27.9-kDa activity detected in *L. lactis* IL1403. The endolysin genes of the three prophages are expressed during growth of *L. lactis* IL1403 and contribute to autolysis. The expression of a similar-sized endolytic activity possibly encoded by homologous genes was detected in the natural strains *L. lactis* subsp. *cremoris* E8, Wg2 and HP that are used in cheese production.

Transcripts of *pi252*, *pi305* and *pi149* were detected by RT-qPCR in *L. lactis* IL1403 but not in *L. lactis* MG1363, which lacks the homologous endolysin genes (Wegmann et al. 2007) and does not produce a ∼30-kDa lytic cell wall hydrolase activity. The analysis of the phage deletion and interruption mutants of *L. lactis* IL1403 shows that all three endolysins contribute to cellular lysis. The 27.9-kDa endolysin activity was present in the cell wall fraction but was not detectable in the supernatant, suggesting that the endolysins, which lack a signal peptide, might be secreted but after translocation are entrapped in the cell wall. The endolysin genes *pi252*, *pi149* and *pi305* are all followed by a gene encoding a pore-forming holin required for secretion of the signal peptide-less phage lysins (Redko et al. 2007). Whether the holin genes are also expressed during growth and contribute to the secretion of the endolysins remains to be investigated. It is also possible that initial lysis is caused by other autolysins such as AcmB or AcmD and that this results in the release of cytoplasmic endolysins. It has been shown that overexpression of phage lysins without their corresponding holins can result in efficient and enhanced cell lysis of *L. lactis* (Steen et al. 2007). Induced expression of the bIL309, bIL285 or bIL286 endolysin in *L. lactis* MG1363*acmAΔ1* resulted in a cumulative effect on lysis. Also, analysis of prophage deletion and insertion mutants of *L. lactis* IL1403 showed that the increased lysis phenotype of IL1403 is the result of the collective activity of the phage lysins.

The fact that the endolysins or their activity was not detectable in the culture supernatant can be explained by the fact that the C-terminal 60 amino acids of the phage lysins of bIL309, bIL285 or bIL286 encompass a putative PG_binding_1 PG binding domain (pfam01471) that has also been identified in other phage lysins (Labrie et al. 2004). A similar type of binding domain in the *Bacillus anthracis* germination-specific lytic enzyme SleB has been shown to bind PG (Heffron et al. 2011).

The identified endolysins seem to be widely present in *L. lactis* strains as was shown for the dairy strains E8, Wg2 and HP. Blast analysis against the GenBank database by using the amino acid sequences of the endolysins encoded by pi252, pi305 and pi149 showed that the genes for homologous endolysins are mostly present in the genomes of *L. lactis* subsp. *lactis*, although they can also be found in genomes from the subsp. *cremoris* and subsp. *lactis* biovar *diacetylactis*. Multiple copies encoding homologues of this endolysin could only be detected in the genome sequences of *L. lactis* subsp. *lactis*. For instance, the human isolate *L. lactis* subsp. *lactis* CV56 contains three homologous phage lysin genes (ADZ64727, ADZ64454, ADZ63676) (Gao et al. 2011), while the genomes of strains SK11 (Makarova et al. 2006) and A12 (Passerini et al. 2013) each contain one homologous endolysin gene (ABJ73565 and CDG04098, respectively). The plant-derived *L. lactis* KF147 strain does not possess any homologous endolysin genes.

A comparison of the PG hydrolytic activity of the highly autolytic *L. lactis* subsp. *cremoris* strain 2250 and its *acmA* deletion derivative revealed that, next to AcmA, extra minor and smaller lytic activities are present that were absent in similar samples from *L. lactis* MG1363 (Riepe et al. 1997). These activities were suggested to act in conjunction with AcmA to cause the lysis phenotype of *L. lactis* 2250. Like the endolysin activities specified by the prophage bIL285, bIL286 and/or bIL309, the cell wall-degrading activity in *L. lactis* 2250 was also shown to be more specific for cell wall fragments of *L. lactis* than those of *M. lysodeikticus*.

Not only did we identify homologous endolysin genes in the natural strains Wg2, E8 and HP, an activity of ∼25 kDa was also present in the cell fractions of the latter two strains. Analysis of the draft genome sequence of *L. lactis* subsp. *cremoris* HP (Lambie et al. 2014) revealed two genes (GenBank EUN34017.1 and EUN33988.1) encoding homologous endolysins with calculated molecular masses of 20.5 kDa that could explain the activity band of ∼25 kDa. This natural expression of phage lysins during growth might be a desired feature for the selection of natural *L. lactis* strains for the production of cheese in order to increase the release of intracellular peptidases needed for enhanced cheese ripening. Screening for these activities might help in the selection of preferred strains for usage in cheese production.

## Electronic supplementary material

ESM. 1 (PDF 201 kb)
