# Supplementary material for: Expression of prophage-encoded endolysins contributes to autolysis of Lactococcus lactis
Source: Appl Microbiol Biotechnol. 2016 Sep 22;101(3):1099–110. doi: 10.1007/s00253-016-7822-z (PMC5247546; doi:10.1007/s00253-016-7822-z)
Supplement: Supplementary file 1 — (PDF 201 kb) [file 253_2016_7822_MOESM1_ESM.pdf]

Applied Microbiology and Biotechnology

Expression of prophage-encoded endolysins contributes to autolysis of *Lactococcus lactis*

Ganesh Ram R. Visweswaran[1*], Dorota Kurek[1], Monika Szeliga[1], Francisco Romero Pastrana[2], Oscar P. Kuipers[1], Jan Kok[1#] and Girbe Buist[1, 2]

[1]Department of Molecular Genetics, Groningen Biomolecular Sciences and Biotechnology Institute (GBB), University of Groningen, Nijenborgh 7, 9747 AG, Groningen, the Netherlands

[2]Department of Medical Microbiology, University of Groningen, University Medical Centre Groningen, Hanzeplein 1, 9700 RB Groningen, the Netherlands

*present address: Department of Immunology, Rikshospitalet, Sognsvannsveien 20, University of Oslo, 0372, Oslo, Norway

[#]**To whom correspondence should be addressed:** (Tel: +31 50 363 2111, Fax: +31 50 363 2348, Email: jan.kok@rug.nl)

Table S1 Primers used in this study

Oligonucleotide*	Sequence; (5'-3')	Restriction Enzyme
FbIL285	CTTCACGAATGGGCCCCAATAGTTATGATTGC	
FbIL286	CCTTGCGAATGGGTCCTAGAAGTTATGAC	
FbIL309	CTTCACGAATGGGTCCAAAAAGTTATGATTGTAG	
RbIl285	TCAGTATTACCCATTGAGCCAGCTGACAG	
RbIL286	TCAGTATTACCCATTGAACCTGCTGACAG	
RbIL309	GTTTCAGTATTACCCATTGAACCTTCTGACAG	
bil286F	ATATGGTCTCCCATGTCAAGTATTGAAAATATGATTGC	*Bsa*I for *Nco*I overhang
bil285F	ATATGGTCTCCCATGCCAAGTATTGAAAATATGATAGC	*Bsa*I for *Nco*I overhang
bil286R	ATATGGTCTCCAGCTTTATAATTTATTCGCATTTAATCGACG	*Bsa*I for *Hin*dIII overhang

*F and R indicate forward and reverse primers of specific endolysin genes, respectively.

Fig. S1

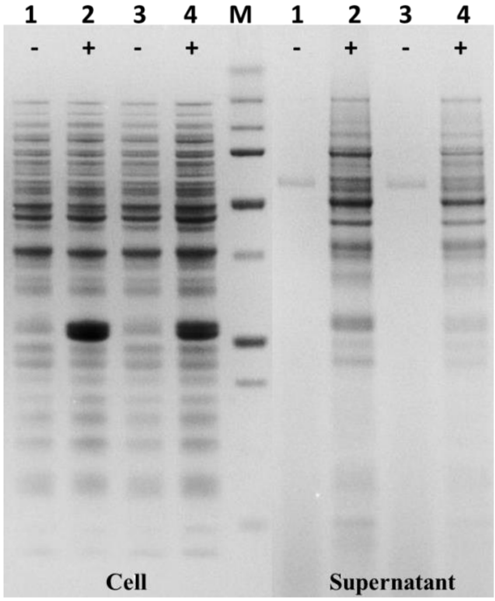

Fig. S1. Detection of overexpression of the endolysins bIL285 and bIL286 in *L. lactis* PA1001. Nisin induced (+) and non-induced (-) culture samples of *L. lactis* PA1001 strains carrying plasmids pNG*bil::pi252* (1, 2) or pNGbil*::pi305* (3, 4) were loaded. Cell (left) and supernatant (right) fractions were loaded. (M), prestained marker (BioRad).
